# Ontario Veterinary Association

**Published:** 1891-02

**Authors:** C. M. Sweetapple

**Affiliations:** Secretary


*Ontario Veterinary Association.*—The annual meeting of the Ontario Veterinary Association was held in the Ontario Veterinary College, Toronto, on December 19th, 1890, at 1:15 P.M. In consequence of the absence of the President, Mr. D. McIntosh, the vice-President, Mr. N. Gibb took the chair, and in opening the meeting made a few appropriate remarks, regarding the absence of the President who had written regretting his not being able to attend. The minutes of the last meeting were then read and confirmed. The Secretary's, Treasurer's and Auditor's reports were then read showing the finances of the association to be in a good condition. The Registrar reported two graduates registering at the last meeting and seventeen since the meeting up to date. The following gentlemen were present at the opening of the meeting, several others came in before its close :

Prof. Smith, Messrs. O'Neil, Quinn, Shaw, J. H. Wilson, J. Wende, Lloyd, Gibb, C. Elliott, W. Cowan. McArthur, Hand, Steele, Hawkins, Gallanough, W. G. Wilson, Heslop, Hopkins, Sweetapple.

Mr. Heslop, of Appleby, Ont., and F. J. Gallanough, of Thornhill, were duly proposed and elected members of the association. Mr. Hopkins, of Detroit, was elected an honorary member of the association. On motion of Prof. Smith seconded by Mr. John Wende, Dr. Huidekoper, of Philadelphia, was elected an honorary member of the association. The payment of dues then took place and in the dissussion on those in arrears it was moved, seconded, and carried that members three years in arrears shall be struck off the roll. J. D. O'Neil, V. S., London, read the following paper on Soundness.

MR. PRESIDENT AND GENTLEMEN :

The subject matter of my paper which I beg to bring before you this evening is that of Examination of Horses as to Soundness ; a subject of the greatest importance to us as Veterinarians, and to the community of horse-owners at large, and one which during all past years has been the cause of much difference of opinion and contention amongst members of our profession, and some of our most eminent authorities.

To young practitioners about to enter upon their professional career or those who have just entered upon it, the subject is always one of interest, for no other test seems to equal that of examining an animal as to soundness ; under may be critical eyes, to a young man with the prospect of world-famed failure if he does wrong and of no world-famed applause if he diagnoses aright. To older practitioners and those of great experience and so accustomed to conducting examinations as to soundness that it becomes as child play * * * The first thing we must decide amongst ourselves is that, of what is to be defined as unsoundness. I take unsoundness to be "That condition of abnomality of an organ or organs, in any part of the animal structure, which interferes with the proper performance of function or functions, or at any time may be liable to cause an interference of same, or to cause an unfitness to perform the purpose for which the animal was intended and consequently depreciates and lessens the value of the animal under examination.

Now having decided what unsoundness is ; how are we to find it out?

D. O'Neil then gave a detailed description of the method of physical examination of the horse, with the diseases, which may effect each part, and continued :

If Veterinary Practitioners will always follow a careful course in their examinations they will make few errors. Now having given my definition of unsoundness and description of my mode of examining for same, we are led up to the question of what really constitues unsoundness, for, on examination we may find blemishes which deteriorate the animal in appearance but not his usefulness, and in no case could really be called an unsoundness. It is our duty to call our clients to the presence of the same, signifying at the same time their harmlessness.

That which really constitutes unsoundness, I take it as any fault or departure from the healthy state, that will come in under my definition of unsoundnsss. Many things that we would draw our client's attention to may not debar the animal from a useful and serviceable life, and he may be practically sound for all general purposes. We say he is practically sound and we *do not* reject the animal, the only result being his value may be depreciated for we *must not indiscriminately reject* every animal for any single defect we see upon him. *Many things* have to be taken into consideration, whether it is likely to permanently effect the animal, the sex, age, temperament and nature of the work of the animal. The extent, nature and position of the defect under notice. But where we come across a case of undoubted unsoundness that is likely to be reproduced in other generations, *then must we reject with a firm, hand* for one and all we should endeavor all we can to put a stop to the transmission of hereditary unsoundness, and, gentlemen, this brings us to another field where we have plenty of scope, for in the list of hereditary unsoundness, I would include opthalmia, cataract, roaring, whistling, ring-bone, side-bone, navicular disease, spavin, curb, grease and shivering, and under some circumstances, bog-spavin, thoroughpin and other tarsal enlargements, as wind galls, splints, weak or contracted feet or stringhalt. So in this formidable list we have plenty of room to exercise our powers upon and I doubt not that many differ upon it now. In like manner as in the past it has always been a bone of contention. I have merely brought before your notice a few of my thoughts in as brief and practical a manner as possible, written with a willing hand in spare moment's by a working practitioner.

Mr. John Wende read a paper on Prolapsus in the bitch.

*Hysterorrhaphy, or Ventral Fixation of the Uterus, For the cure of inversion of that organ in the bitch.*

On October 24, 1890 I was called to attend a Newfoundland bitch, eleven months old, said to have a large swelling under her tail. Upon examination I found the uterus inverted, the tumor being about the size of two fists, covered with pus and emitting an offensive odor. The hairs of the tail and hind leg's were matted together, making the animal a very loathsome object.

The owner told me that the bitch had come into heat about three weeks previous, and she, being young, and never having been bred before, he decided not to stint her to a dog until the latter part of the oestrum. On the sixth or seventh day he noticed her straining, and also the appearance of the tumor in the vulva. She continued her explusive efforts, and in twenty-four hours the protruding organ had increased to the size of a man's fist. He then consulted an Empiric, who prescribed a powder to be given internally, and the parts to be swabbed with equal parts ot warm water and milk several times daily. He also cautioned the owner not to touch the organ with his hands, as it might produce "blood poisoning" to both animal and man.

After employing the prescribed treatment for two weeks, during which time the dog assumed the appearance above described, the owner was advised to consult me. I had her brought to my infirmary, and with the assistance of my student, R. H. McMullen, washed the parts thoroughly, placed her on a table under the influence of ether, removed all scabs, and by continued pressure for one hour, succeeded in returning the protruding viscus to its natural position. We then stitched up the vulva with four interrupted stitches of linen tape, fed the patient on a laxative diet, and washed the parts twice daily, using a syringe and antiseptic and astringent lotions, composed of water, boracic acid, alum and sulphate of zinc alternately. This treatment was continued for one week without any apparent improvement, as the organ persisted in resting against the stitches in the vulva, even if it were placed into position with a pessary a dozen times daily.

I then decided on the above named operation, which has been successfully performed on human subjects, for the details of which I would refer the reader to two articles by Howard Kelly, of Philadelphia. The first in *the American Journal of Obstetrics, Vol. XX., Jan. 1887*, and the second in *the American Journal of the Medical Sciences, May 1888*. Also to one by Sänger on *Operative Treatment of Retroversio-flexio Uteri, in the Centralblatt für Gynäcologie, Nos. 2 and 3, 1888*. It was as follows: Again placed the bitch on the table under ether, shaved the hair from the posterior part of the abdomen, made an incision about five inches long on the linea alba between the last four teats, passed my hand into the abdomen, grasped the uterus, and by gentle traction drew it forward into position, and fastened it about one inch above the incision with three interrupted stitches of heavy catgut. In inserting the needle I aimed to grasp about one-third of the body of the uterus, and quite deeply into the muscles of the abdominal wall. I then sponged out the abdomen with a weak solution of boracic acid, sutured the muscles with catgut, the common integument with linen thread, removed the stitches from the vulva, the muzzle from the nose, dashed a little cold water on the head and chest of the patient, and in a few moments she stood up apparently none the worse from the effects of the ordeal. On Nov. 6, seven days after the operation I removed the stitches from the skin and sent the bitch home all right, with the exception of the wound on the abdomen, which was about three inches long, and that she evinced pain whenever she tried to sit down upon her haunches for three weeks after the operation. But this trouble has gradually worn off, and at present she is as well as ever.

It was moved by Mr. Wilson, seconded by Prof. Smith and carried that the thanks of the meeting be presented to the readers of papers. And the Secretary was instructed to forward them to the JOURNAL OF COMPARATIVE MEDICINE AND VETERINARY ARCHIVES, and to the Veterinary Journal of London England.

Dr. Duncan mentioned instances of nodules on the mesentery produced by parasitic worms.

Mr. Hawkins described some cases of furunculus or gangrene, in Detroit, and cases of the same, were mentioned as having occurred in Toronto, Buffalo, and some other cities. Mr. Gibb mentioned that the disease had occurred in Boston in 1857 and had been there treate with pyroligneous acid.

Dr. Duncan suggested that the sloughing might have been produced by lowered vitality similar to that produced by ergot, but in these cases produced by cold.

Prof. Smith suggested that cold and slush, perhaps salt on snow or ice, sometimes put on to keep the tracks of street cars clear, may possibly produce it, but he believed it due to some local poison probably a micro organism, and he advocated the use of strong antiseptics, such as carbolic acid, he did not reccommend poulticing.

It was moved, seconded and carried that the Secretary should procure some more copies of the tariff and also get out a printed list of the Registered graduates.

Mr. Gibb vacated the chair and Mr. Cowan presided.

The election of officers then took place with the following result:—

Mr. W. Gibb, St. Mary's, President; Mr. D. McArthur, Ailsa Craig, First vice-President; Mr. J. Wende, Buffalo, N. Y., V. S., Second vice-President; Mr. C. N. Sweetapple, Toronto, Secretary; Mr. W. Cowan, Galt, Treasurer; Messrs. O'Neil and Elliott, Auditors; Messrs. Burns, Hand, W. H. Wilson, Steele, Gallanough, Hopkins, Ormsby and Lloyd, Directors.

Messrs. J. H. Wilson and O'Neil, Delegates to Western Fair Association

Mr. W. Cowan, Delegate to Central Permanent Farmers' Institute.

Prof. Smith was elected Honorary President.

Mr. Hawkins spoke in disapproval of qualified practitioners associating themselves in business with empirics. He also mentioned that many prominent positions were occupied in the United States by Graduates of the Ontario Veterinary College, and was greatly in favor of the Summer practice which is required of all students who attend this college,

It was moved seconded and carried, that the sum of $25.00 be appropriated for a medal to be competed for by the students of the Ontario Veterinary College, at the approaching Spring examinations. The usual fee of $50.00 was then voted to the secretary for his services.

Dr. Duncan remarked that Prof. Axe disapproved of the operation in consequence of the severity of the cough that supervened. Prof. Smith said that the general opinion amongst veterinary surgeons in Great Britain was not in favor of the operation. But that it might perhaps be beneficial in some cases for slow work.

At the close of this discussion the meeting adjourned.

C. M. SWEETAPPLE, *Secretary.*

